# Toxicity of Pristine and Chemically Functionalized Fullerenes to White Rot Fungus *Phanerochaete chrysosporium*

**DOI:** 10.3390/nano8020120

**Published:** 2018-02-22

**Authors:** Zhu Ming, Shicheng Feng, Ailimire Yilihamu, Qiang Ma, Shengnan Yang, Sheng-Tao Yang

**Affiliations:** College of Chemistry and Environment Protection Engineering, Southwest Minzu University, Chengdu 610041, China; 15982889615@163.com (Z.M.); 18328594054@163.com (S.F.); almira1250@outlook.com (A.Y.); qiang9322@outlook.com (Q.M.); 18641460881@163.com (S.Y.)

**Keywords:** fullerene, white rot fungi, growth inhibition, structural change, degradation activity, nano-biosafety

## Abstract

Fullerenes are widely produced and applied carbon nanomaterials that require a thorough investigation into their environmental hazards and risks. In this study, we compared the toxicity of pristine fullerene (C_60_) and carboxylated fullerene (C_60_-COOH) to white rot fungus *Phanerochaete chrysosporium*. The influence of fullerene on the weight increase, fibrous structure, ultrastructure, enzyme activity, and decomposition capability of *P. chrysosporium* was investigated to reflect the potential toxicity of fullerene. C_60_ did not change the fresh and dry weights of *P. chrysosporium* but C_60_-COOH inhibited the weight gain at high concentrations. Both C_60_ and C_60_-COOH destroyed the fibrous structure of the mycelia. The ultrastructure of *P. chrysosporium* was changed by C_60_-COOH. Pristine C_60_ did not affect the enzyme activity of the *P. chrysosporium* culture system while C_60_-COOH completely blocked the enzyme activity. Consequently, in the liquid culture, *P. chrysosporium* lost the decomposition activity at high C_60_-COOH concentrations. The decreased capability in degrading wood was observed for *P. chrysosporium* exposed to C_60_-COOH. Our results collectively indicate that chemical functionalization enhanced the toxicity of fullerene to white rot fungi and induced the loss of decomposition activity. The environmental risks of fullerene and its disturbance to the carbon cycle are discussed.

## 1. Introduction

Since their discovery, carbon nanomaterials have been developed and hold great potential in diverse areas [1]. Fullerenes, nanotubes, graphene, carbon quantum dots, nanodiamonds, and carbon nanoparticles are widely investigated and produced nowadays [2,3,4,5,6]. During their production and applications, it is inevitable that these carbon nanomaterials would have contact with the environment. Therefore, their environmental risks and toxicity are attracting great interest nowadays [7,8,9,10]. Fullerenes are among the most attractive carbon nanomaterials due to their precisely defined structures, unique physical and chemical properties, and various important applications [11]. Fullerenes are applied in many areas, such as electronics [12], energy [13], biomedicine [14], environment [15], and so on. The sp^2^ carbon cages of fullerenes have much higher chemical reactivity than carbon nanotubes and graphene. When fullerenes enter the environment, chemical oxidation of fullerenes occurs, which changes their environmental behaviors and bio-effects [16]. Thus, both the pristine and functionalized fullerenes should be investigated in the environmental hazard evaluations.

The literature indicates that fullerenes and their derivatives are toxic to organisms in the environment, which is regulated by the chemical functionalization and/or dispersion reagents [17,18,19,20]. In particular, the interaction between fullerene and fungi is attracting attention from the community [21]. For example, fullerenols (C_60_-OH) slightly reduced mycelial biomass weight but significantly decreased aflatoxin concentration in media, which was due to the antioxidative activity of C_60_-OH within fungal cells [22]. More *Aspergillus niger* spores formed after the 120 h exposure to C_60_-OH [23]. Wang et al. found that fullerene C_60_ did not affect the antifungal activity of *Fusarium graminearum* and *Fusarium poae* [24]. Despite the influence of fullerene on fungi, the biotransformation of fullerene by fungi was also observed. Schreiner et al. reported that white rot fungi could degrade fullerenols after a 32-week incubation [25]. However, the current knowledge for understanding the fungi–fullerene interaction and its environmental effects is limited.

Recently, the impact on the carbon cycle has been realized as an essential issue in the environmental risk evaluations of carbon nanomaterials [26,27]. The carbon cycle is critical for the earth’s habitability by humans and other large fauna [28]. The core issue of the carbon cycle is the interconversion between inorganic and organic carbon forms that maintain the CO_2_ balance in the atmosphere [29]. CO_2_ enters the biosphere through photosynthesis, while the organic carbon forms leave the biosphere via three pathways: the decomposition by microorganisms, the breathing of living creatures, and the burning of debris. During the decomposition, the white rot fungi convert wood into humus that subsequently decomposes into CO_2_, which returns to the atmosphere. White rot fungi produce laccase (Lac), manganese peroxidase (MnP), and ligninase for the decomposition, which could also be used for pollution remediation [30]. Several studies have shown that carbon nanomaterials can influence the decomposition capability of white rot fungi. Xie et al. reported that graphene oxide (GO) inhibits the growth of white rot fungi and leads to the complete loss of degradation activity for dye [27]. Yang et al. suggested that chemical reduction alleviates GO effects on white rot fungi [31]. After reduction by vitamin C, reduced GO (RGO) does not suppress the degradation activity of white rot fungi. Similarly, RGO hydrogels and xerogels were found to immobilize and enhance the Lac production of white rot fungus *Trametes pubescens* [32]. In addition, protein level studies have indicated that carbon nanotubes and graphene influence the enzyme activity of white rot fungi [31,33]. For fullerene, its impact on the decomposition of white rot fungi has not been evaluated yet, which hinders the full understanding of the environmental safety of fullerene.

**Scheme 1:**
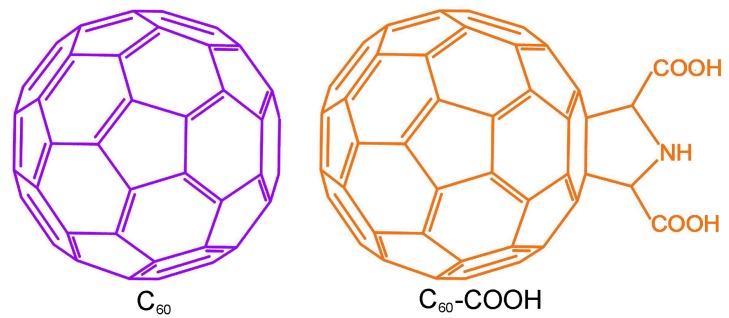
Structure of C_60_ and C_60_-COOH.

In this study, we evaluated the toxicity of pristine and carboxylated fullerene C_60_ (Scheme 1) to white rot fungus *Phanerochaete chrysosporium* and quantified the decomposition activity changes. The growth of *P. chrysosporium* was investigated by measuring the dry and fresh weights of mycelia. The structural changes were observed by optical microscopy, scanning electron microscopy (SEM), and transmission electron microscopy (TEM). The enzyme activities of Lac and MnP were quantified. The decoloration of the dye reactive brilliant red X-3B and the degradation of sawdust were evaluated to reflect the impact on the decomposition activity of *P. chrysosporium*. The importance of chemical functionalization and the implication to the environmental safety evaluation of carbon nanomaterials are discussed.

## 2. Materials and Methods 

### 2.1. Materials

Fullerene C_60_ (declared purity of >99.5 wt %) was purchased from Shenzhen Nanotech Port, Ltd., Shenzhen, China. The carboxylation of C_60_ was strictly performed following our previous report [18]. Both C_60_ and C_60_-COOH were characterized by TEM (Tecnai G2 20, FEI, Hillsboro, OR, USA), X-ray photoelectron spectrum (XPS, Kratos, Manchester, UK), infrared spectroscopy (IR, Magna-IR 750, Nicolet, Alexandria, LA, USA), and Raman spectroscopy (inVia, Renishaw, Wotton-under-Edge, UK) before use.

### 2.2. Toxicity Evaluations

A fungal strain *P. chrysosporium* (ACCC 30942) was obtained from the Agricultural Culture Collection of China. The culture of fungi was performed using the same protocols in our previous report [27].

To investigate the toxicity of fullerene, the culture medium was supplemented with C_60_ or C_60_-COOH at concentrations of 0–1.0 mg/mL for *P. chrysosporium* culture. The pH values of the culture media were adjusted to 4.5 with NaOH or HCl aqueous solutions (PB10, Sartorius Co., Gottingen, Germany) before exposure. Each flask was inoculated with 40 mL medium containing 2.0 × 10^7^ spores. The flasks were shaken on a thermostat shaker (CHA-S, Jintan Hankang Electronic Co., Jintan, China) at 150 rpm and 37 °C for 14 d before toxicity measurements. 

For growth inhibition investigation, the mycelia of *P. chrysosporium* were filtered and the pH values of the filtrate were measured by a pH meter. The extra water was absorbed by filter paper and the weight of *P. chrysosporium* was measured (fresh weight). The mycelia were dried in a vacuum oven at 110 °C for 24 h and weighed again (dry weight). For IR analyses, the filtered fresh mycelia were lyophilized. Each 1.0 mg sample was mixed with 100 mg KBr for pelleting. The IR spectra of the samples were recorded on the IR spectrometer.

For morphology observations, the filtered fresh mycelia were fixed with 4% glutaraldehyde solution overnight. Standard techniques were applied to prepare the paraffin sections for periodic acid-Schiff (PAS) staining. The PAS-stained sections were investigated under a light microscope equipped with a charge-coupled device (CAB-30PC, Cabontek Co., Chengdu, China). For SEM observations, the filtered fresh mycelia were lyophilized for 3 d and coated with gold for 5 s in the chamber of a sputter coater (JFC 1_60_0, JEOL, Tokyo, Japan). The gold-coated samples were observed under a SEM (S-4800, Hitachi, Tokyo, Japan). For ultrastructural changes, the filtered fresh mycelia were fixed by 2.5% glutaraldehyde overnight and post-fixed in 1% osmium tetroxide. The fixed samples were dehydrated in a graded alcohol series and embedded in epoxy resin for sectioning on an ultramicrotome. Thin sections were post-stained with uranyl acetate and lead citrate for TEM observations.

### 2.3. Enzyme Activities

For enzyme activity measurements, *P. chrysosporium* were exposed to C_60_ or C_60_-COOH for 14 day and filtered for the filtrate collection as described in the toxicity evaluations. The Lac activity was measured using 2,2′-azino-bis(3-ethylbenz-thiazoline-6-sulfonate) (ABTS) as the substrate [34]. The filtrate was diluted with the buffer to allow the proper kinetics according to premeasurements. Then, 1.0 mL of diluted filtrate, 0.2 mL of 0.5 mM ABTS and 2.7 mL of 0.1 M sodium acetate buffer (pH 4.8) were mixed and the absorbance at 420 nm was immediately monitored on a UV-VIS spectrometer (UV1600, Shanghai Mapada Instruments Co., Shanghai, China). The initial slope was used for the Lac activity calculation.

For the MnP activity assay, the aforementioned filtrate was diluted to allow the proper kinetics according to premeasurements. Then, 0.4 mL of diluted filtrate was added with 0.1 mL of 1.6 mM MnSO_4_ (substrate) and 3.4 mL of 50 mM sodium lactate buffer (pH 4.5). The reaction was triggered by adding 0.1 mL of 1.6 mM H_2_O_2_. The absorbance at 240 nm was monitored and the initial slope was obtained for MnP activity calculation [35].

### 2.4. Degradation Capabilities

For the decoloration of dye reactive brilliant red X-3B, the medium was supplemented with C_60_ or C_60_-COOH (0–1.0 mg/mL) and inoculated with 5.0 × 10^5^ spore/mL. The flasks contained 40 mL inoculated media for each and were shaken at 150 rpm at 37 °C for 3.5 day. The reactive brilliant red X-3B solution (1.23 mg/mL, Shanghai Citailong Co., Ltd., Shanghai, China) was sterilized by filtration through a 0.22 μm membrane and introduced to the flasks at a final concentration of 30 mg/L. The flasks were incubated for another 2.5 day for decoloration and the absorbance of the supernatant was measured at 538 nm for the decoloration efficiency calculation. 

For the degradation of wood, the sawdust (Dashuo Experimental Animal Co., Chengdu, China) was sterilized at 121 °C for 15 min before inoculation. The dry sawdust (5.0 g) was mixed with 12 mL of solid fermentation medium (supplemented with 0–1.0 mg/mL of C_60_ or C_60_-COOH). The sawdust was inoculated with 2.0 × 10^7^ spores and maintained static at 37 °C for 90 d. Separately, the sterilized sawdust without inoculation with spores was used as the non-degraded sample. At 90 day, the sawdust samples were washed with deionized water to remove residual mycelia and dried to constant weights at 105 °C. The dry weight of sawdust was measured for degradation percentage calculation. The sawdust samples were also washed and dried overnight at 45 °C for SEM and IR analyses.

### 2.5. Statistical Analysis 

The data are expressed as the average mean of individual observations with standard deviation (mean ± SD). Significance was calculated by the Student’s *t*-test method. The difference was considered as statistically significant at *p* < 0.05 and indicated by ^*^ for the comparison between the fullerene-exposed group and the control group, and ^#^ for the comparison between the C_60_ group and the C_60_-COOH group of the same exposure concentration.

## 3. Results and Discussion

### 3.1. Influence of Fullerene on Fungus Growth

**Figure 1:**
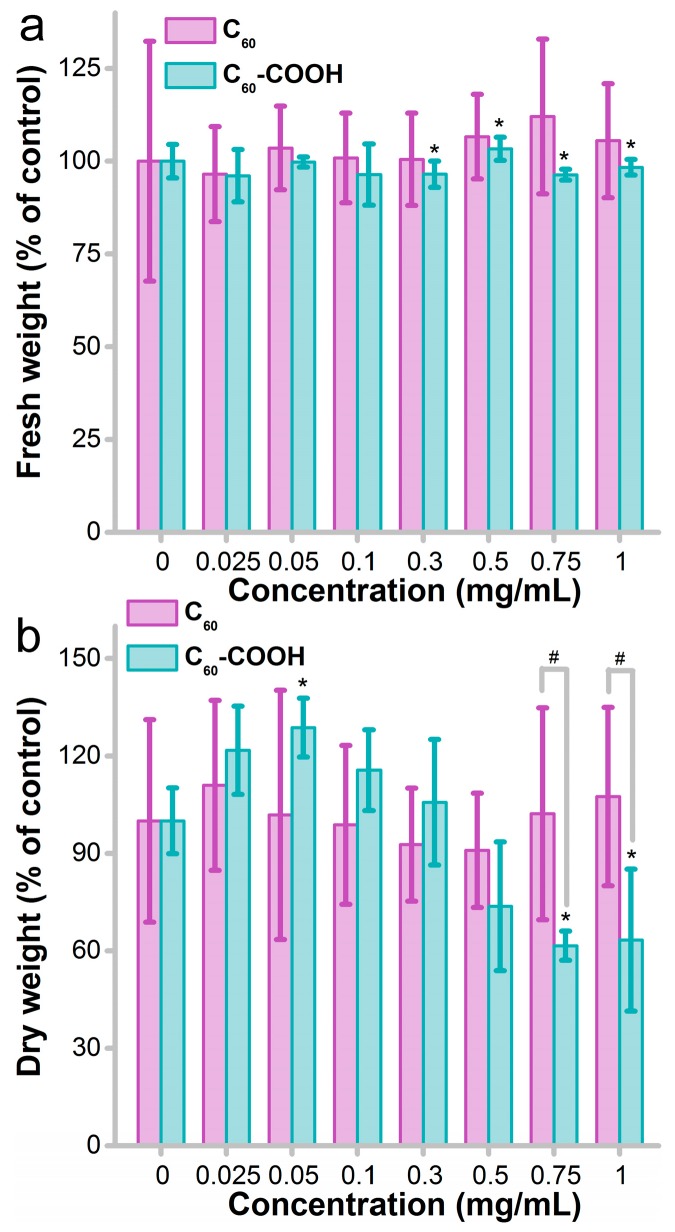
Influence of fullerene on the weight gain of *P. chrysosporium*. (**a**) Fresh weight; (**b**) dry weight. * *p* < 0.05 compared to the control group; # *p* < 0.05 comparing C_60_ and C_60_-COOH.

Pristine fullerene C_60_ is a dark brown powder that hardly disperses in aqueous systems. After the functionalization of the carboxyl groups, C_60_-COOH becomes water dispersible to form a brown dispersion. The characterization data were consistent with our previous report (Figures S1–S3) [18]. The C_60_ and C_60_-COOH were introduced to *P. chrysosporium* after sonication. As shown in Figure 1a, the fresh weights of *P. chrysosporium* were not seriously influenced by either C_60_ or C_60_-COOH. Although the statistic changes were found at C_60_-COOH concentrations of 0.3–1 mg/mL, the fresh weights were in the range of 96.4–103.4% of the control. On the other hand, the dry weight was significantly stimulated to 129% of control at a C_60_-COOH concentration of 0.05 mg/mL, which was attributed to hormesis. Serious inhibition of dry weights was observed at C_60_-COOH concentrations of 0.75 mg/mL (62% of control) and 1.0 mg/mL (63% of control). No hazardous effect of pristine C_60_ was observed based on the fresh and dry weight measurements. The difference between C_60_ and C_60_-COOH was significant when analyzing the dry weights at exposure concentrations of 0.75 and 1.0 mg/mL, suggesting that functionalized C_60_ was more toxic. When monitoring the pH values of the culture systems, C_60_ did not influence the pH of the culture media (Figure S4a) but C_60_-COOH led to a mild acidification of the media (Figure S4b).

The inhibition of weight gain of white rot fungi by nanomaterials has been widely reported in the literature. Previously, we found that GO stimulated the growth of *P. chrysosporium* at 1.2 mg/mL (*p* < 0.05) after a 14-d incubation and largely inhibited the weight gain at 2 mg/mL and higher (*p* < 0.05). This was consistent with this report [27]. Upon chemical reduction, RGO stimulated the fresh weight gain of *P. chrysosporium* at 0.25 mg/mL and higher. A meaningful dry weight stimulating effect was observed at RGO concentrations of 1.0 mg/mL and higher [31]. Huang et al. reported that Fe_3_O_4_ nanoparticles (NPs) slightly inhibited the biomass gain of *P. chrysosporium* at 0.5 mg/mL after 1 d exposure and stimulated the biomass gain at day 3 [36]. It should be noted that for both graphene and fullerene, the chemical functionalization increased their toxicities according to the gain in biomass. A possible reason might be that the chemical oxidation increased the dispersibility of the carbon nanomaterials, which led to higher contact between the carbon nanomaterials and *P. chrysosporium*. Similarly, in the protein–graphene interaction study, more dispersible GO had a higher influence on the lysozyme activity and conformation [37].

### 3.2. Structural Changes upon the Exposure to Fullerene

**Figure 2:**
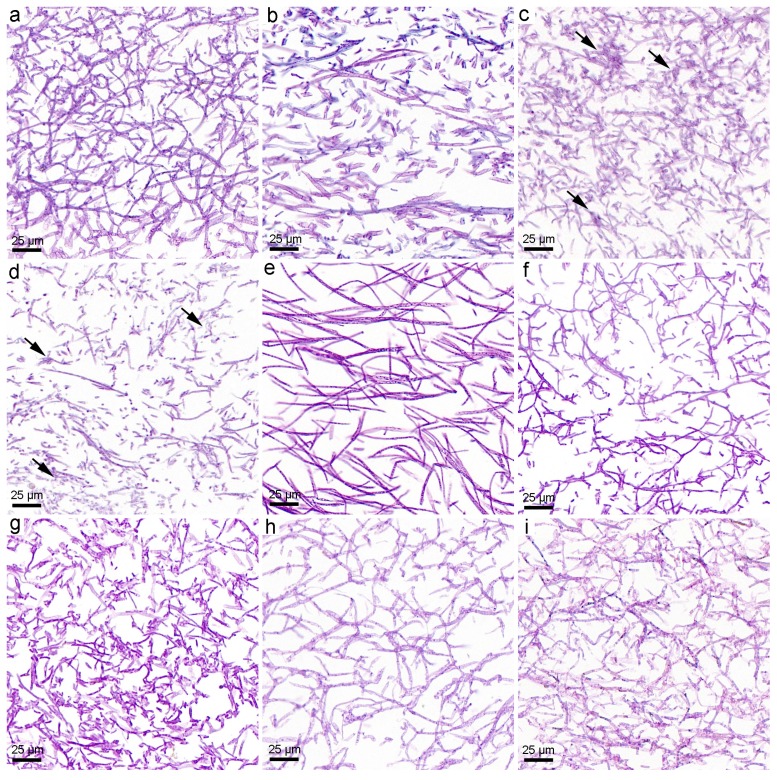
Images of periodic acid-Schiff (PAS)-stained *P. chrysosporium*. (**a**) Control; (**b**) 0.05 mg/mL of C_60_; (**c**) 0.3 mg/mL of C_60_; (**d**) 0.75 mg/mL of C_60_; (**e**) 1.0 mg/mL of C_60_; (**f**) 0.05 mg/mL of C_60_-COOH; (**g**) 0.3 mg/mL of C_60_-COOH; (**h**) 0.75 mg/mL of C_60_-COOH; (**i**) 1.0 mg/mL of C_60_-COOH. The amorphous aggregates of mycelia are indicated as arrows in (**c**,**d**).

Typically, *P. chrysosporium* had fibrous mycelia that formed pellets. Without adding fullerene, very good fibers were presented under optical microscopy after PAS staining (Figure 2a), where the glycogen of mycelia was oxidized by periodic acid and stained with Schiff reagent. With the addition of C_60_, the mycelia became shorter and thicker even at the low concentration of 0.05 mg/mL (Figure 2b). The lengths of the mycelia were even shorter at 0.3 and 0.75 mg/mL and had some amorphous aggregates (Figure 2c,d). Surprisingly, the mycelia recovered at a C_60_ concentration of 1 mg/mL (Figure 2e). The mycelia seemed longer than those of the control. C_60_-COOH had a similar influence as C_60_ on the fibrous structures of *P. chrysosporium* mycelia (Figure 2f,g). The fibers recovered at C_60_-COOH concentrations of 0.75 and 1.0 mg/mL (Figure 2h,i).

**Figure 3:**
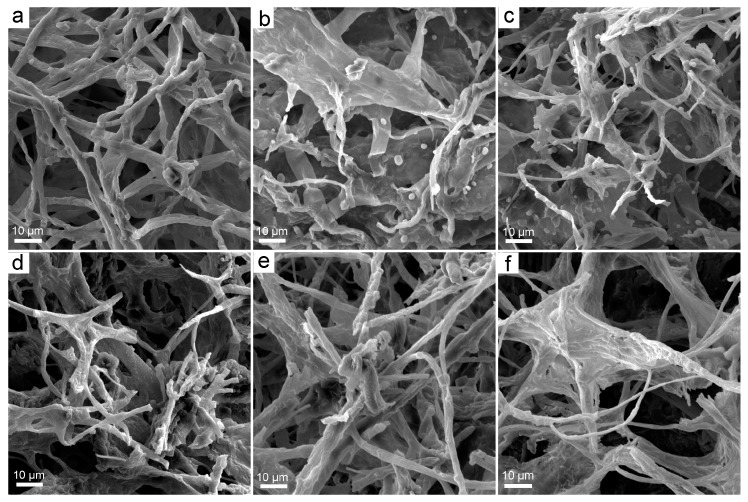
SEM images of *P. chrysosporium*. (**a**) Control; (**b**) 0.3 mg/mL of C_60_; (**c**) 1.0 mg/mL of C_60_; (**d**) 0.3 mg/mL of C_60_-COOH; (**e**,**f**) 1.0 mg/mL of C_60_-COOH.

SEM provided a better observation of the mycelia of *P. chrysosporium*. The smooth fibers of the control group were clearly recognized (Figure 3a). When exposed to C_60_ at 0.3 mg/mL, the fibrous structure of *P. chrysosporium* was destroyed (Figure 3b). The mycelia became flat and short. The morphology had the appearance of ribbons or even sheets rather than fibers. Some small cubes were found on the mycelia, which were the inorganic salts. At 1.0 mg/mL of C_60_, more fibers were found, consistent with the PAS results. The sheet-like structure was still recognizable, suggesting a disturbance in the mycelia formation. The changes in the fibrous structure were milder for C_60_-COOH-exposed *P. chrysosporium*. The fibers of the mycelia became shorter at 0.3 mg/mL of C_60_-COOH (Figure 3d). They were more stick-like, which was consistent with the optical microscopic observations. At 1.0 mg/mL, the long fibers recovered (Figure 3e) and only a few were sheet-like (Figure 3f).

**Figure 4:**
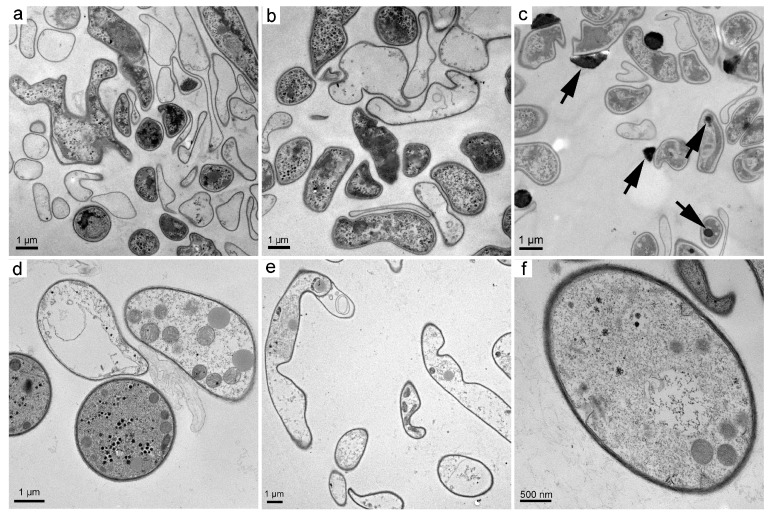
TEM images of *P. chrysosporium*. (**a**) Control; (**b**) 0.3 mg/mL of C_60_; (**c**) 1.0 mg/mL of C_60_; (**d**) 0.3 mg/mL of C_60_-COOH; (**e**,**f**) 1.0 mg/mL of C_60_-COOH. The attached and entering C_60_ aggregates are indicated by the arrows.

The ultrastructure of *P. chrysosporium* was investigated under TEM. Small round cells and some irregular cells are presented in the TEM image of the control group (Figure 4a). The sizes of these fungus cells were about 1–2 μm in diameter. At a C_60_ concentration of 0.3 mg/mL, the ultrastructure of *P. chrysosporium* was not influenced (Figure 4b). At a high C_60_ concentration of 1.0 mg/mL, many C_60_ aggregates were attached to the fungus cells and a few of them entered the cells (Figure 4c). Detachment of the cell wall and membrane was observed, suggesting toxicity of C_60_ to *P. chrysosporium*. At 0.3 mg/mL, C_60_-COOH did not change the ultrastructure of the fungus cells (Figure 4d). At 1.0 mg/mL, most cells had lost the cell plasma but the cell wall seemed intact (Figure 4e). Some vesicles containing small particles were also observed (Figure 4f).

The disturbance of mycelia by nanomaterials is a widely reported phenomenon. At a GO concentration of 0.1 mg/mL, the fiber lengths of the mycelia decreased and the amorphous structures increased. The ultrastructure of *P. chrysosporium* changed significantly upon exposure to GO, where the cell membrane and wall were clearly broken. The SEM images indicate the rolling of mycelia into balls surrounded by GO [27]. Upon exposure to RGO at 0.25 mg/mL, the mycelia length and thickness increased [31]. The mycelia became amorphous at an RGO concentration of 4.0 mg/mL. The shape of the cells became irregular and the cell membrane and wall became fuzzy under TEM. The fibers became thicker and broadened at low RGO concentrations but the widths of the mycelia decreased at an RGO concentration of 4.0 mg/mL. Another study on CdSe/ZnS quantum dots (QDs) indicate that the morphology of *P. chrysosporium* became incompact and granular after the exposure [38]. In Huang et al.’s study, widened mycelia loaded with some crystal particles were observed after exposure to Ag NPs [39]. Overall, it can be found that upon exposure to nanomaterials, the morphological changes of *P. chrysosporium* were inevitable and irrespective of whether growth inhibition occurred. Nanomaterials have a large surface area and tended to bind to the mycelia of *P. chrysosporium* after incubation, thereby possibly disturbing the mycelia structure.

### 3.3. Impact of Fullerene on Enzyme Activity

**Figure 5:**
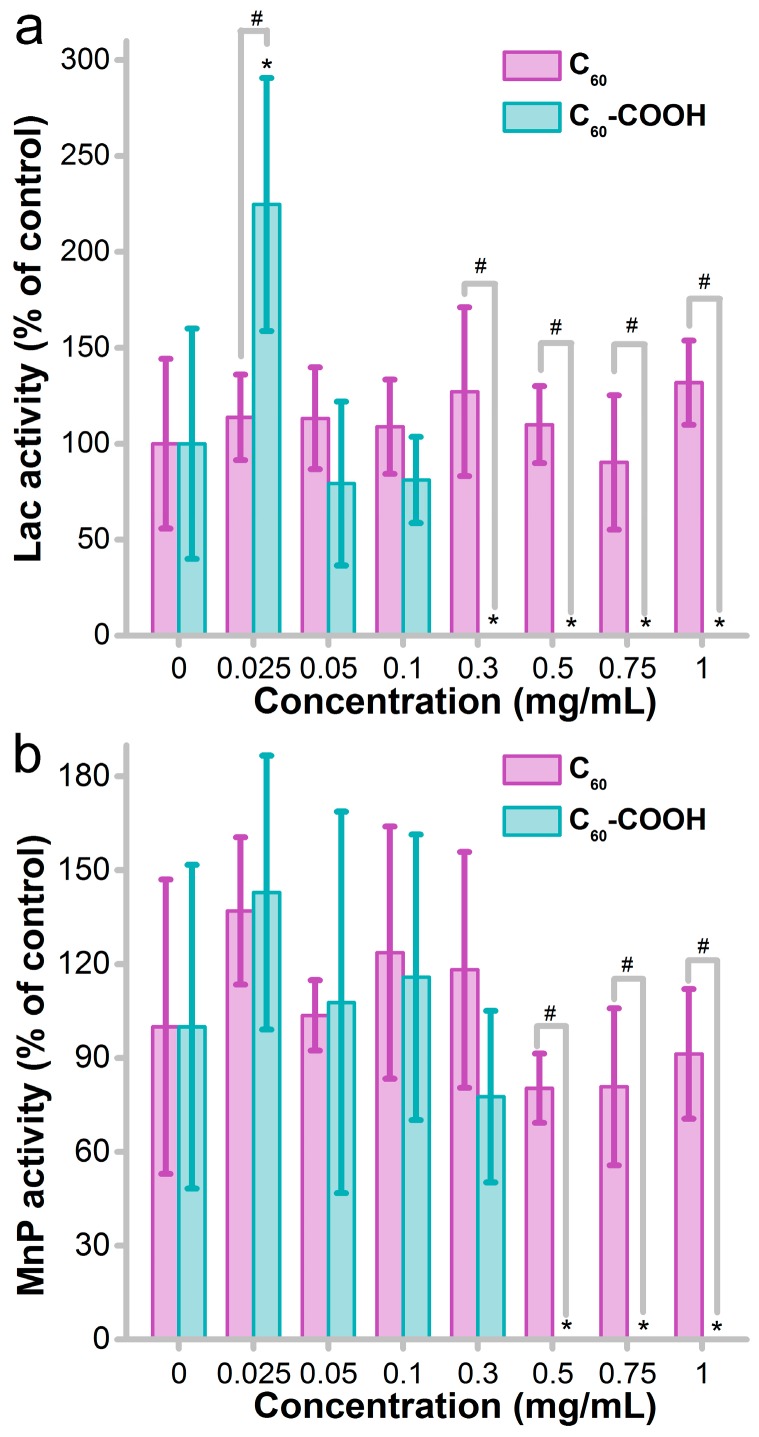
Influence of fullerene on the laccase (Lac) activity (**a**) and manganese peroxidase (MnP) activity (**b**) of *P. chrysosporium*. * *p* < 0.05 compared to the control group; # *p* < 0.05 comparing C_60_ and C_60_-COOH.

The production of oxidative enzymes is the most important function of white rot fungi for oxidizing and degrading the lignin and organic pollutants. Here we quantified the enzyme activities of Lac and MnP to reveal the impact of fullerene. As indicated in Figure 5a, pristine C_60_ did not influence the activity of Lac in the concentration range of 0–1.0 mg/mL. C_60_-COOH had a stimulating effect on Lac activity of *P. chrysosporium* at 0.025 mg/mL. Complete loss of Lac activity was observed at C_60_-COOH concentrations of 0.3 mg/mL and higher. The difference between C_60_ and C_60_-COOH was also significant at 0.3 mg/mL and higher, suggesting the inhibition of Lac activity by functionalized fullerene. For MnP, no impact of pristine C_60_ was found during our test (Figure 5b). The complete loss of MnP activity occurred at C_60_-COOH concentrations of 0.5 mg/mL and higher. The difference was significant between C_60_ and C_60_-COOH at 0.5 mg/mL and higher. The loss of enzyme activity in the liquid culture system implies that functionalized fullerene leads to the complete loss of degradation capability.

Protein–nanomaterial interaction is one of the fundamental issues of nano-bioeffects [40]. The binding of protein with fullerene was mainly due to the aromatic rings that had π-π interaction with the aromatic residues of the protein [41]. The protein–fullerene interaction has been well documented in our previous reports as the functionalization regulated interaction that led to the conformational changes and enzyme activity loss [41,42]. For white rot fungi, several studies have focused on the enzyme activity after exposure to nanomaterials. We found that RGO did not change the Lac activity of *P. chrysosporium* [31]. Rodriguez-Couto found that RGO hydrogel did not affect the Lac activity of *T. pubescens* and RGO xerogel significantly stimulated the Lac production [32]. Berry et al. reported that both unpurified metal catalyst-rich carbon nanotubes (CNTs) and purified carboxylated CNTs promoted significant changes in the oxidative enzyme activity of white rot fungi, while pristine CNTs did not [33]. Shah et al. found that Cu NPs decreased the Lac production by *Trametes versicolor* but did not change the MnP production [43]. Fe NPs did not alter the Lac and MnP activity of *T. versicolor*. Due to the low enzyme amount, the current studies did not distinguish the inhibition of enzyme activity by nanomaterials and the inhibition of protein expression. Nevertheless, it could be seen that the influence of nanomaterials on the enzyme activity of white rot fungi depended on the chemical components and functionalization.

### 3.4. Influence of Fullerene on the Decomposition Activity

**Figure 6:**
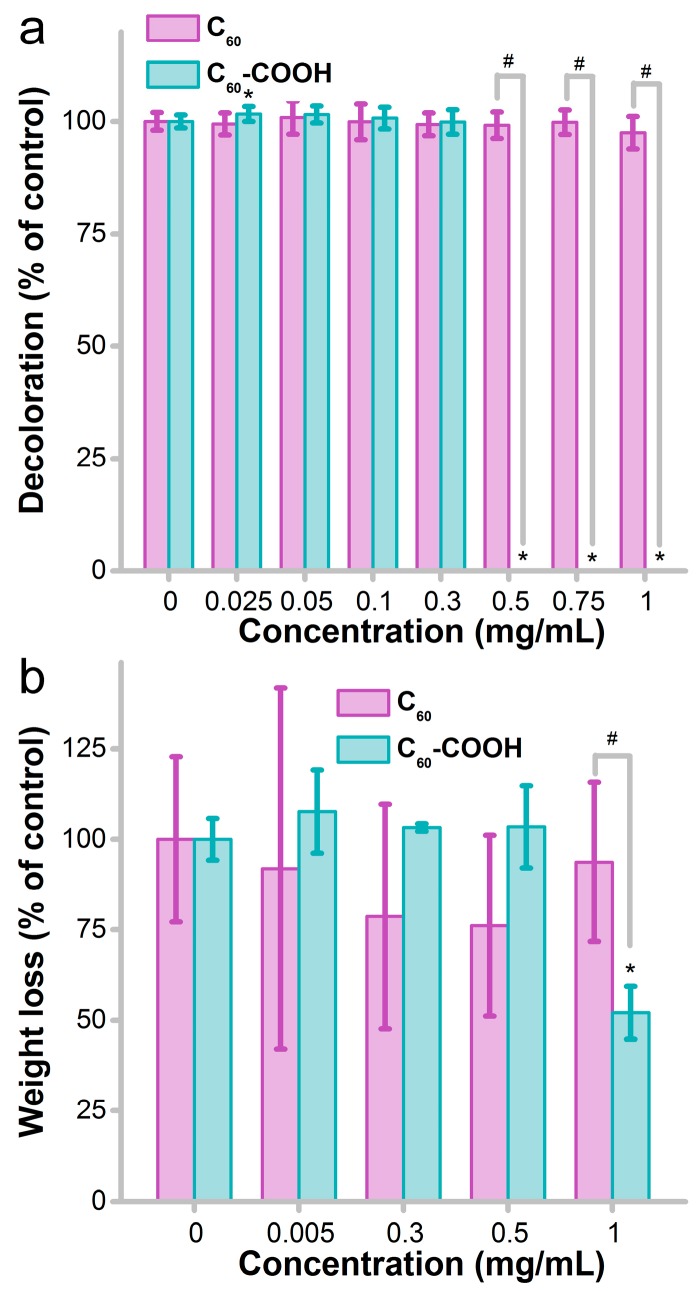
Influence of fullerene on the decoloration of reactive brilliant red X-3B (**a**) and the degradation of sawdust (**b**) by *P. chrysosporium*. * *p* < 0.05 compared to the control group; # *p* < 0.05 comparing C_60_ and C_60_-COOH.

As decomposers, the most important ecological function of white rot fungi is to decompose the organic remains and, in particular, wood and straws. Two typical experiments were performed to evaluate the influence of C_60_ on the decomposition activity of *P. chrysosporium*. Firstly, we tested the decoloration of the classical substrate of white rot fungi, namely reactive brilliant red X-3B. As shown in Figure 6a, the decoloration efficiency of reactive brilliant red X-3B was nearly identical for the control and C_60_-exposed groups. No significant difference was observed (*p* > 0.05). C_60_-COOH seemed to inhibit the decoloration at high C_60_-COOH concentrations, except the tiny enhancement at a C_60_-COOH concentration of 0.025 mg/mL. The loss of degradation capability upon the exposure to C_60_-COOH was consistent with the enzyme activity loss in Figure 5. Generally, the decoloration of dye by white rot fungi could be due to three contributors: oxidation by *P. chrysosporium* enzymes, the adsorption by *P. chrysosporium* mycelia, and the adsorption effect of C_60_. The overall decoloration was not influenced, suggesting that C_60_ did not influence the detoxification of pollutants.

**Figure 7:**
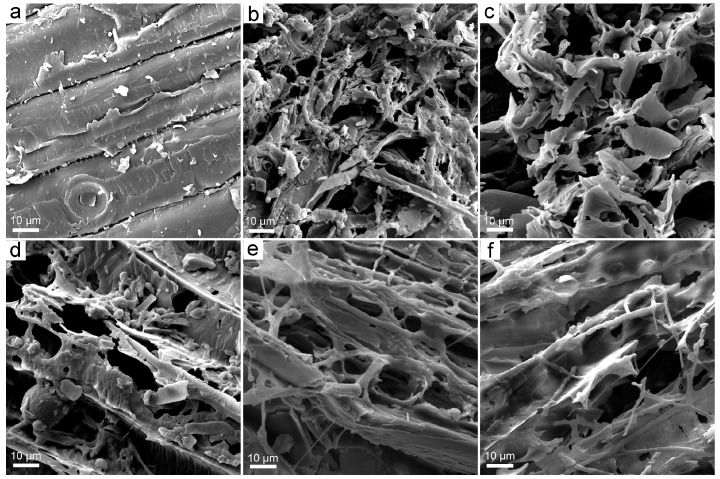
SEM images of sawdust before and after degradation by *P. chrysosporium*. (**a**) Non-degraded sawdust; (**b**) control; (**c**) 0.3 mg/mL of C_60_; (**d**) 1.0 mg/mL of C_60_; (**e**) 0.3 mg/mL of C_60_-COOH; (**f**) 1.0 mg/mL of C_60_-COOH.

For sawdust degradation, there was no adsorption involved. The degradation was carried out as the static fermentation. As shown in Figure 6b, the decomposition activity of *P. chrysosporium* was significantly suppressed by C_60_-COOH after a 90-d incubation at a high concentration (1 mg/mL). The inhibition rate of sawdust weight loss was 48% at 1 mg/mL of C_60_-COOH compared with that of the control group. On the other hand, C_60_ had no significant influence on the degradation of sawdust. The difference between C_60_ and C_60_-COOH was significant at 1.0 mg/mL. The weight loss rates of sawdust indicate that C_60_-COOH had a higher impact on the decomposition function of white rot fungi and might block the carbon cycle at the decomposition link. To verify the decomposition capability loss, we scanned the surface morphology of sawdust to directly compare the degradation degree. The untreated sawdust had a nearly intact surface (Figure 7a). As shown in Figure 7b, in the control group, the sawdust had a broken surface under SEM, indicating the efficient degradation of the wood. Attached mycelia on the sawdust surface are clearly presented. Upon exposure to C_60_, a well-degraded surface can also be observed (Figure 7c,d). Some spores and mycelia are observed alongside the detached fragments. The surface degradation also occurred in the C_60_-COOH-exposed group (Figure 7e,f). The sawdust surface is relatively less degraded in Figure 7f, which was exposed to 1.0 mg/mL of C_60_-COOH, suggesting less efficient decomposition.

The decomposition activity is the main ecological function of white rot fungi. When nanomaterials interact with white rot fungi, the decomposition activity might be changed. Previously, we found that RGO did not change the decomposition activity in decolorizing reactive brilliant red X-3B and degrading sawdust [31]. Li et al. reported that Fe_2_O_3_ NPs facilitated the degradation of bisphenol A by *Pleurotus ostreatus* via the Fenton reaction [44]. Huang et al. found that the oxalate–Fe_3_O_4_ system could promote the fungus decomposition of phenol under light [36]. Filpo et al. found that TiO_2_ NPs prevented fungi growth in wood and the corresponding degradation [45]. Taghiyari et al. found that Ag and Cu NPs prevented particleboard degradation by *T. versicolor* fungus [46]. Akhtari et al. used IR to investigate the degradation of Paulownia fortune by *T. versicolor* fungus and found fungicidal effects of Ag, Cu, and ZnO NPs [47].

### 3.5. Implications

White rot fungi are the main microorganisms that decompose lignin and are thus crucial in maintaining the carbon cycle. According to our results, when fullerenes enter the environment, the fullerene–fungus interaction might lead to the loss of decomposition activity of the white rot fungi at high fullerene concentrations, thus threatening the carbon cycle and the ecological balance. It should be noted that the inhibition concentrations of fullerene here were much higher than the environmental concentrations according to the literature [48,49], suggesting the relative safety of fullerenes. Serious hazards might occur only when fullerenes are accidentally released into the environment. In addition, chemical functionalization strongly affects the impact of fullerenes on white rot fungi. Water-soluble fullerene interacts more strongly with the fungi and has higher hazards. Thus, the release of dispersible fullerene into the environment deserves more attention. However, the biotransformation of fullerene in the environment is inevitable and could be oxidized into dispersible formulations, in particular under light irradiation [50]. The influence of biotransformation on the environmental safety of fullerenes should be carefully studied in the future.

Our results indicate the importance of chemical functionalization on fullerene toxicity to white rot fungi. As previously mentioned, carboxylated fullerene had higher toxicity to white rot fungi and led to the complete loss of the decomposition activity at high concentrations. Pristine fullerene was generally less toxic with only morphological changes being observed upon exposure to pristine fullerene. A similar phenomenon has been observed with graphene, where oxidized graphene was much more toxic than the reduced form [27,31]. Clearly, the reduced forms of both fullerene and graphene are non-dispersible in aqueous systems, which largely reduces the direct contact with white rot fungi. The reduced exposure certainly would alleviate the environmental risk of carbon nanomaterials. Beyond the dispersibility, the pristine fullerene has lower protein interactions, according to the literature [51]. Pristine fullerene does not influence the enzyme activity and conformation according to the report from literature [52,53,54]. On the other hand, functionalized fullerene seriously inhibits lysozyme activity and induces the loss of secondary structure [42]. Therefore, from the perspective of protein–fullerene interaction, the lower toxicity of fullerene is also reasonable. Such information is well summarized and discussed in a recent review [55]. 

Mechanistically, the toxicity of fullerenes to white rot fungi should be related to the oxidative stress. Oxidative stress is widely reported in the toxicity evaluations of nanomaterials [56]. Ag NPs and CdSe/ZnS QDs induce oxidative stress in white rot fungi according to reports from Zeng et al. [38,39]. Fullerenes have been found to induce oxidative stress in various biological systems [57,58], although fullerenes have also usually been regarded as antioxidative reagents [59]. Further investigations are required in the future to clarify the toxicological mechanisms.

## 4. Conclusions

In summary, chemical functionalization largely enhanced the toxicity of fullerene to white rot fungi, while pristine fullerene was less toxic. C_60_-COOH inhibited the fungus growth according to the weight gain and seriously disturbed the mycelium structure. The complete loss of decomposition activity was observed in the liquid culture of *P. chrysosporium* in the presence of high concentrations of C_60_-COOH. The decrease in degradation activity induced by C_60_-COOH was confirmed in the fermentation of sawdust. Since pristine fullerene is likely oxidized after entering the environment, both pristine and functionalized fullerenes might induce hazards to white rot fungi and hold a potential threat to the decontamination by white rot fungi and threaten the carbon cycle. It is hoped that our results would stimulate more interest in the environmental risks and safe applications of carbon nanomaterials.

## Supplementary Materials

The following are available online at http://www.mdpi.com/2079-4991/8/2/120/s1. Figure S1. Representative TEM images of C_60_ (a) and C_60_-COOH (b). Figure S2. C1s XPS spectra of C_60_ (a) and C_60_-COOH (b). Figure S3. Raman spectra of C_60_ (a) and C_60_-COOH (b). Figure S4. The pH values of the *Phanerochaete chrysosporium* culture systems before and after the incubation for 14 d with C_60_ (a) and C_60_-COOH (b). * *p*<0.05 comparing to the control group.
