# Supplementary material for: Toxicity of Pristine and Chemically Functionalized Fullerenes to White Rot Fungus Phanerochaete chrysosporium
Source: Nanomaterials (Basel). 2018 Feb 22;8(2):120. doi: 10.3390/nano8020120 (PMC5853751; doi:10.3390/nano8020120)
Supplement: Supplementary file 1 [file nanomaterials-08-00120-s001.zip › SI-C60-WRF.docx]

**Supplementary materials**

**
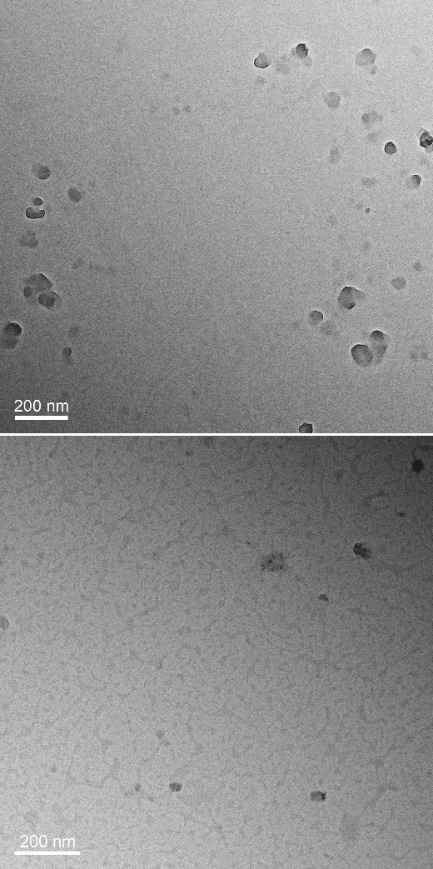
**

**Figure S1.** Representative TEM images of C_60_ (a) and C_60_-COOH (b).

**
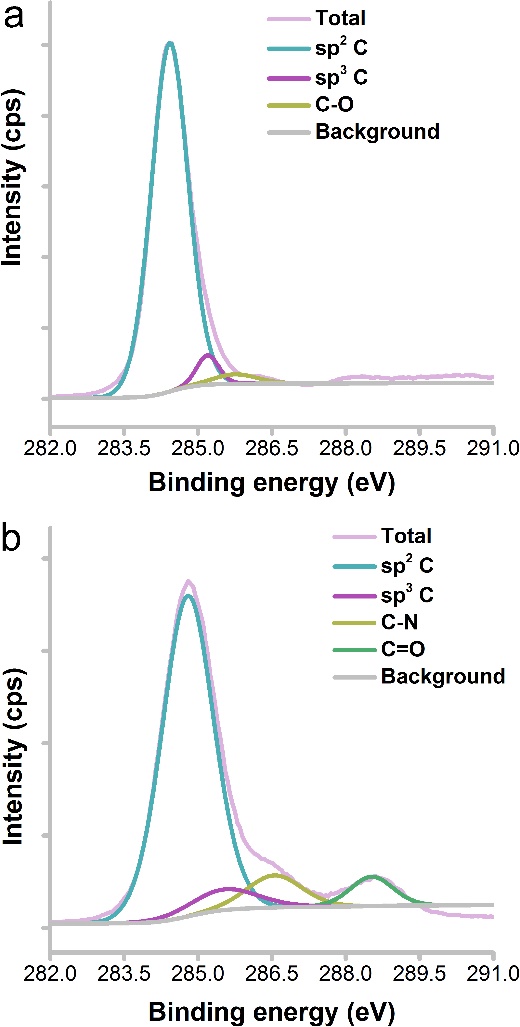
**

**Figure S2.** C1s XPS spectra of C_60_ (a) and C_60_-COOH (b).


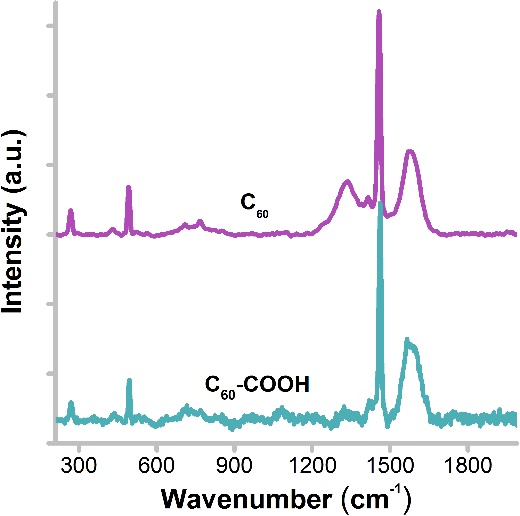


**Figure S3.** Raman spectra of C_60_ (a) and C_60_-COOH (b).

**
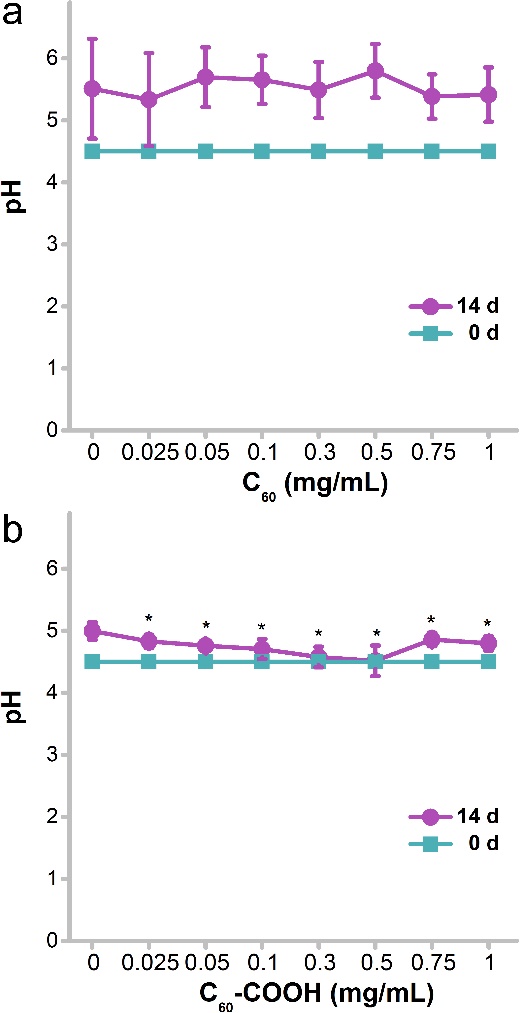
**

**Figure S4.** The pH values of the *Phanerochaete chrysosporium* culture systems before and after the incubation for 14 d with C_60_ (a) and C_60_-COOH (b). * *p*<0.05 comparing to the control group.
